# The Impact of Protocol Assignment for Older Adolescents with Hodgkin Lymphoma

**DOI:** 10.3389/fonc.2014.00317

**Published:** 2014-11-28

**Authors:** Richard S. Pieters, Henry Wagner, Stephen Baker, Karen Morano, Kenneth Ulin, Maria Giulia Cicchetti, Maryann Bishop-Jodoin, Thomas J. FitzGerald

**Affiliations:** ^1^Department of Radiation Oncology, University of Massachusetts Medical School, University of Massachusetts Memorial Health Care System, Worcester, MA, USA; ^2^Division of Radiation Oncology, Milton S. Hershey Medical Center, Pennsylvania State University, Hershey, PA, USA; ^3^Department of Quantitative Health Sciences and Cell Biology, University of Massachusetts Medical School, Worcester, MA, USA; ^4^Department of Radiation Oncology, Quality Assurance Review Center, University of Massachusetts Medical School, Lincoln, RI, USA

**Keywords:** adolescents, young adults, Hodgkin lymphoma, radiotherapy, clinical trials

## Abstract

**Background and Purpose:** Hodgkin lymphoma (HL) treatment has evolved to reduce or avoid radiotherapy (RT) dose and volume and minimize the potential for late effects. Some older adolescents are treated on adult protocols. The purpose of this study is to examine the protocol assignment of older adolescents and its impact on radiation dose to relevant thoracic structures.

**Materials and Methods:** Cooperative group data were reviewed and 12 adolescents were randomly selected from a pediatric HL protocol. Treatment plans were generated per one pediatric and two adult protocols. Dose volume histograms for heart, lung, and breast allowed comparison of radiation dose to these sites across these three protocols.

**Results:** A total of 15.2% of adolescents were treated on adult HL protocols and received significantly higher radiation dosage to heart and lung compared to pediatric HL protocols. Adolescents treated on either pediatric or adult protocols received similar RT dose to breast.

**Conclusion:** Older adolescents treated on adult HL protocols received higher RT dose to thoracic structures except breast. Level of nodal involvement may impact overall RT dose to breast. The impact of varying field design and RT dose on survival, local, and late effects needs further study for this vulnerable age group. Adolescents, young adults, Hodgkin lymphoma, RT, clinical trials

## Introduction

Hodgkin lymphoma (HL) affects patients of all ages, particularly adolescents and young adults (ages 16–34). Historically, radiotherapy (RT) to all involved lymph node volumes was the first available curative treatment for children and adults. Eventually, two schools of treatment philosophy evolved. One favored subtotal nodal irradiation, defined as treating the nodes in the neck, axillae, mediastinum (the traditional mantle field), plus an abdominal field encompassing spleen, the para-aortic, and pelvic nodes. The other was more tailored and allowed for treatment of only the mantle field after staging laparotomy and splenectomy (1). Doses for both regimens were 40–44 Gy. With the advent of chemotherapy (CTX), the RT doses decreased slightly. As recently as the 1990s, either subtotal nodal or mantle irradiation to 36–40 Gy was still administered following CTX (2).

The Quality Assurance Review Center (QARC) has been a National Cancer Institute (NCI) supported resource, providing RT quality assurance for several of the NCI Cooperative Groups performing cancer clinical trials (3). With the NCI transformation of the Cooperative Group program in March 2014, QARC is now part of the Imaging and Radiation Oncology Core Group and is known as IROC RI. During the course of the protocols investigated in this report, RT data were evaluated at QARC to ensure compliance with Cooperative Group protocol specifications (4). In the course of performing RT reviews, it became clear that older adolescents were being treated on both pediatric and adult protocols, for unstated reasons, but presumably due to protocol criteria or institutional priorities. Adolescents are known to have similar outcomes to pediatric patients, but their management varied according to the protocol being followed.

As survival improved, concern shifted to minimizing the late effects, particularly for children, on growth, vital organs, and carcinogenesis. Patient management has evolved to include risk and response driven adaptive therapy using anatomic and metabolic imaging (5). In order to avoid the late effects of both therapies, low-risk patients receive only CTX and are not irradiated on either adult or pediatric protocols. Although this strategy is commonly used, until protocol data matures, it remains investigational. For intermediate risk patients, the pediatric protocols now utilize a lower dose of radiation, 21 Gy, with CTX (5). This strategy is embedded in pediatric trials but influences management of adults to a lesser degree. A recent pediatric protocol, COG AHOD0031, randomized patients achieving rapid early response and a complete response (CR) to no RT vs. low dose involved field irradiation. Similar trials of CTX-only strategies in low-risk adult patients showing early metabolic CR to initial CTX are maturing, with early results showing a higher risk of recurrence if RT is omitted but without differences in survival (6, 7).

Given the variation in treatment strategy and the known importance of dose delivered to normal organs on risk of late effects, we decided to explore further the issue of protocol assignment for late adolescents and young adults, aged 16–21 years. The first objective was to ascertain the proportion of patients assigned to either a pediatric or adult protocol. The next objective was to examine the impact of protocol specified radiation regimens on dose to lung, heart, and breast. It was hypothesized that, for patients requiring RT, the treatment plans specified on these pediatric protocols would deliver significantly less RT to relevant thoracic normal structures, heart, lung and, for females, breast, than adult protocols. The final objective was to discuss the relationship between dose, late effects, survival, and factors that should be taken into consideration before treatment assignment as well as directions for further research in this important area.

## Materials and Methods

Cooperative group HL data were reviewed to identify adolescent patients, aged 16–21 years, based on the youngest patients allowed on the adult trials (≥16 years) and the oldest on the pediatric trials (≤21 years). The pediatric trials were POG 9425, POG 9426, CCG59704, COG AHOD0031, AHOD03P1, and AHOD0431. The adult trials included were SWOG 9133 (an example of subtotal nodal/mantle treatment, although treatment data was not in the QARC database), ECOG 2496, SWOG 9901, and CALGB 50203. The first of these trials opened in 1992 and the last closed in 2010 (Table 1). The choice of protocol varied, apparently dependent on institutional priorities, treating medical oncology service, or available open protocols in a particular facility. CTX regimens were diverse in agents and duration. Each patient on protocol had consented to the use of their data as part of the IRB process approved at each treating institution.

**Table 1:** **Protocol dates and patient numbers**.

Trial	Open dates	Total patients	Patients RI	Patients NoRT	Patients (16–21 years)	Patients (16–21 years) RT	Patients (16–21 years) NoRT
**PEDIATRIC**							
POG 9426	10/1996–10/2005	293	262	31	81	77	4
POG 9425	03/1997–03/2001	173	158	15	55	52	3
CCG 59704	10/1999–05/2001	9S	56	42	30	21	9
COG AHOD0031	09/2002–10/2009	1715	1176	539	660	462	198
COGAHOD03P1	01/2006–11.2010	1S7	11	176	44	3	41
COGAHOD0431	02/2006–04/2009	277	120	157	105	56	49
Total PEDI		2743	1783	960	975	671	304
**ADULT**							
SWOG 9133	09/1992–04/2000	348	348	0	45	45	0
ECOG 2496	04/1999–06/2006	855	512	343	118	81	37
Arm A		429	180	249	59	27	32
Arm B		426	332	94	59	54	5
SWOG 9901	04/2000–11/2001	11	0	11	1	0	1
CALGB 50203	05/2004–09/2006	98	0	98	11	0	11
Total ADULT		1312	860	452	175	126	49
Total combined		4055	2643	1412	1150	797	353

Because COG AHOD0031 (pediatric trial) required submission of pretreatment diagnostic images and treatment portals for pre-review, and closed shortly after the advent of digital imaging, a limited number of digital image sets were available for review. Twelve patients with complete digital image sets were randomly selected for analysis. COG AHOD0031 was the largest of the pediatric trials and required involved field radiation therapy (IFRT) for all patients except for those who achieve a rapid early response by volumetric criteria (CT or MRI) after two cycles of CTX AND a CR after four cycles of CTX. These patients were randomized to receive or not receive IFRT.

**Figure 1:**
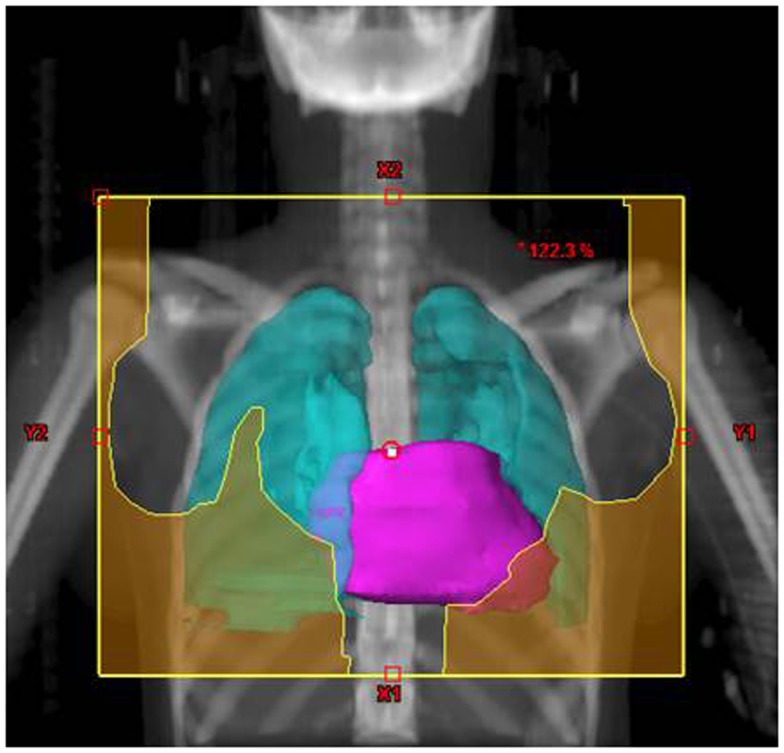
**Treatment field definitions for AHOD0031**.

**Figure 2:**
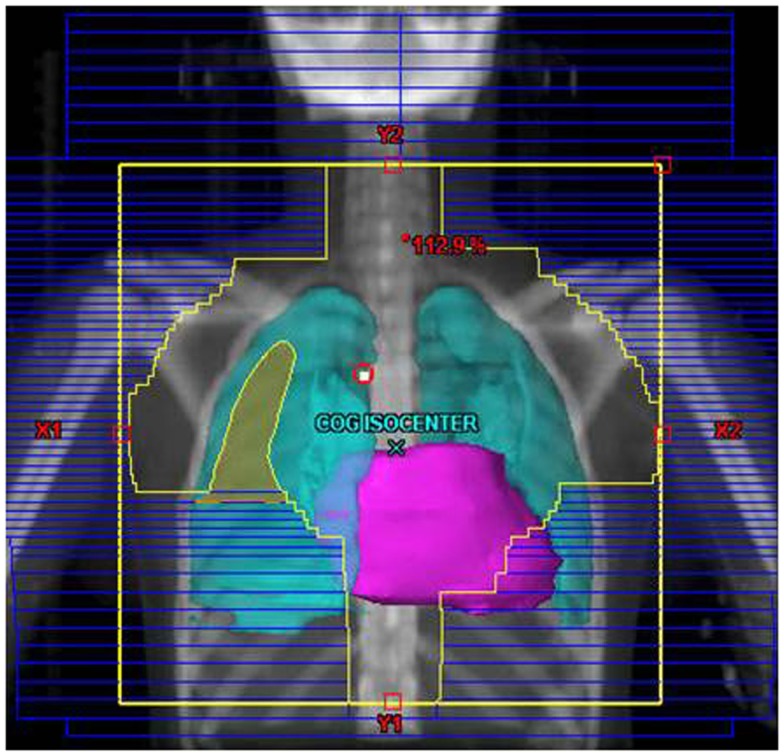
**Treatment field definitions for ECOG 2496 Arm B, planned on COG protocol image set**.

**Figure 3:**
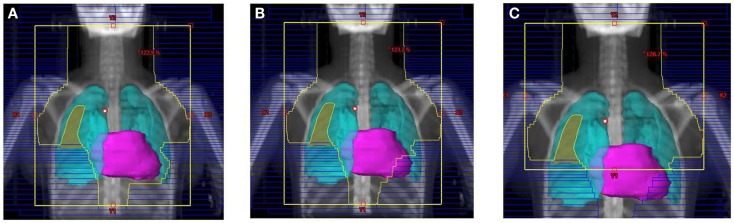
**Treatment field for the SWOG 9133 protocol for patient #4: (A) initial volume to 14 Gy; (B) first volume reduction to 30 Gy; (C) final volume reduction to 36 Gy**.

Their plans had been reviewed at QARC for compliance with protocol guidelines. Ten patients, seven females and three males, had stage I or II (supra-diaphragmatic) disease and one male and one female had stage III disease. Treatment plans for these adolescents were reviewed to examine the impact of treatment variation on dose to three normal structures: lung, heart, and breast. Figure 1 shows the field definitions for AHOD0031. It was decided the radiation doses, these 12 patients actually received on the pediatric trial would be compared to the doses they would have received had they been assigned to two adult trials, ECOG 2496 (Figure 2) and SWOG 9133 (Figure 3). SWOG 9133 used subtotal nodal radiation 36–40 Gy, but only the traditional mantle portion that delivered dose to the thoracic organs was evaluated. The ECOG adult protocol provided IFRT, 36 Gy/20 fractions, for patients with bulk disease at presentation, defined as >5 cm for the Stanford V arm (Arm B), or to the mediastinum for tumor measuring >1/3 chest diameter for the ABVD arm (Arm A). All 12 of the selected patients met the criteria of 5 cm, disease in the mediastinum, and would have required RT on ECOG Arm B, but only 4 (including 3 females) had mediastinal disease >1/3 chest diameter and would have required RT on ECOG Arm A. Therefore, it was decided to exclude Arm A from the analysis due to insufficient statistical power (Table 2).

**Table 2:** **Selected protocol eligibility criteria and details of protocol specified RT**.

Trial	Age eligibility	Stage eligibility	Radiation (RT) dosages and volumes
**PEDIATRIC**			
POG 9426	0–21 years	Stage: I, IIA (including IIIA1), disease limited to spleen, splenic, celiac, or portal nodes	Involved field RT: 25.5 Gy
POG 9425	0–21 years	IIB, IIB LMA, IIIB, IV	RT dosage: 21 Gy
			Stage l and II: mantle RT
			Stage III and IV: subtotal or total nodal RT
CCG 59704	< 21 years	Stage: IIB. IIIB	RT dosage: 21 Gy
		IV B	RER males: involved field radiotherapy, RER females, no RT
			Slow early response (SER): involved field RT
COG AHOD0031	0–21 years	Bulk disease only: IA, IIA	CR Pts: randomized, NFT vs. Involved field RT 21 Gy (liver 15 Gy, solid organs 10.5 Gy)
		±Bulk disease: IB, IIB, IAE, IIAE, IIIAS, IIIAE + S:IVA, IVAE	VGPR, PR stable Pts: involved RT, as above
COG AHOD0431	0–21 years	Non-bulk disease (−lymphocyte predominant): IA and IIA	IFRT: 21 Gy
COG AHOD03P1	0–21 years	Non-bulk disease (+lymphocyte predominant): IA and IIA	IFRT: 21 Gy
**ADULT**			
SWOG 9133	≥ 16 years	IA. IEA. IIA, IIEA. no laparotomy, no infra-diaphragmatic disease	Subtotal nodal radiotherapy (mantle + spleen/para-aortic sequential): 36–40 Gy
ECOG 2496	≥ 16 years	Locally extensive: I–IIA/B, III, IV	Arm A: 36 Gy, only to initial mediastinal disease, to residual disease pre-chemo length by post-chemo width
			Arm B: 36 Gy, to bulky disease sites (>5 cm) +macroscopic splenic disease; to residual disease pre-chemo length by post-chemo width
SWOG 9901	≥ 15 years	III or IV	No radiotherapy
CALGB 50203	≥ 16 years	IA, IB, IIA, IIB, except nodular lymphocyte predominant	No radiotherapy

### Treatment planning modeling

De-identified pretreatment diagnostic imaging and treatment planning CT scans were imported into the Varian Eclipse treatment planning system. The original COG AHOD0031 treatment plans were reconstructed in Eclipse and plans for the adult protocols were developed on the same CT scan sets in accordance with the respective protocol guidelines. All these protocols specified opposed treatment beams to treat the designated target volumes; 3-dimensional conformal treatment, intensity modulated RT (IMRT), and volumetric modulated arc therapy (VMAT) techniques were not permitted.

The tumor volumes, protocol specified clinical target volumes, normal lung and heart, and, for the females, breast volumes were contoured. For each patient, a plan was generated for each protocol, COG AHOD0031, SWOG 9133, and ECOG 2496 Arm B. Each of these plans underwent standard QARC review to confirm protocol compliance. For the two IFRT protocol arms, it was elected to plan the same fields for the pediatric 21 Gy and the adult 36 Gy, to assess the impact of prescribed dose on the dose to normal organs of interest (breast, lung, and heart) within each arm. As total nodal or standard mantle therapy is no longer a component of protocol therapy, these fields were not recalculated for the lower dose. Dose volume histograms (DVHs) were generated and the mean breast, heart, and lung doses, the V20 for lung and V5 for breast, heart and lung were recorded for each plan. Treatment fields for a typical patient with axillary involvement for the AHOD0031 and ECOG Arm B protocols are shown in Figures 1 and 2; this patient required axillary treatment on both IFRT arms. SWOG 9133 treatment fields are shown in Figure 3.

### Statistical methods

The difference in dose to the normal structures was evaluated using analysis of variance by fitting general linear mixed models (8) (a form of ANOVA for repeated measures). Models were fit by restricted maximum likelihood estimation (9) using the SAS Proc Mixed procedure (10). In the presence of significant differences among means, pairwise comparisons were made using Tukey’s HSD multiple comparisons procedure (11) utilizing the estimated covariance matrix to account for correlated observations for analysis of paired comparisons. The distributional characteristics of outcome measures were evaluated by applying the Kolmogorov–Smirnov Goodness of Fit Test for Normality (12) to residuals from fitted linear models and by inspection of frequency histograms of these residuals. In some cases, natural logarithms of outcomes were applied to better approximate normally distributed residuals. All computations were performed using the SAS version 9.2 (13) and SPSS Version 19 (14) statistical software packages. Statistical significance is defined as present when associated *p*-values are <0.05. Differences with *p*-values between 0.05 and 0.10 were described as “approaching significance.”

## Results

Between 1992 and 2010, 1150 patients between the ages of 16 and 21 were treated on North American cooperative group HL protocols, 975 (84.8%) on pediatric protocols, and 175 (15.2%) on adult protocols. These patients constituted 35.5% of patients enrolled on these pediatric protocols and 28.3% of patients on these adult protocols. Of the 1150 patients, 353 (30.7%) were treated with CTX alone, and 797 (69.3%) with RT, as specified. Of the 975 patients on pediatric protocols, 671 (68.8%) were treated with RT. Of the 175 patients on adult protocols, 126 (72.0%) were treated with RT. There was no statistically significant difference in the percentage of patients treated with RT in the pediatric vs. adult protocols (*X*^2^ = 0.69, *p* > 0.10).

Table 3 demonstrates the impact of protocol specified fields at constant dose on normal tissue dose. Table 3A shows that the SWOG traditional mantle component of the required subtotal nodal plan delivered significantly more RT dose to heart, lung, and breast tissue than the ECOG 2496 Arm B adult trial or the pediatric trial at 36Gy. There were no significant differences between ECOG 2496 Arm B and the pediatric trial at 36 Gy for doses to heart, lung, or breast. Similarly, Table 3B compares Arm B at 21Gy to pediatric trial at 21Gy. Again, there were no significant differences in doses delivered to heart, lung, and breast when each prescription was reduced to 21Gy. Within each dose (21 or 36 Gy), the ECOG 2496 Arm B and COG AHOD0031 protocols, with differently defined involved field plans, delivered similar doses to heart, lung, and breast.

**Table 3:** **(A) Impact of treatment arm on normal tissue dose for 36 Gy prescription; (B) impact of treatment arm on normal tissue dose for 21 Gy prescription**.

	Mantle	Arm B (adult)	Pediatric	Mantle vs. Arm B	Mantle vs. Pediatric	Arm B vs. Pediatric
**(A)**						
Heart (mean dose) in Gy	29.8	20.2	19.4	9.6 *p* < 0.0001	10.4 *p* < 0.0001	0.8 *p* = 0.992 (n.s.)
Lung (V20) in percent	51.7	35.1	42.8	16.6 *p* < 0.0001	8.9 *p* < 0.0001	−7.7 *p* = 0.100 (n.s.)
Breast (mean dose) in Gy	12.0	7.0	8.5	5.0 *p* < 0.0009	3.4 *p* = 0.0387	−1.5 *p* = 0.790 (n.s.)

	**Arm B (adult)**	**Pediatric**	**Arm B vs. Pediatric**			

**(B)**						
Heart (mean dose) in Gy	11.8	11.5	0.2 *p* = 1.000 (n.s.)			
Lung (V20) in percent	21.4	26.0	−4.6 *p* = 0.556 (n.s.)			
Breast (mean dose) in Gy	4.1	5.1	−1.1 *p* = 0.939 (n.s.)			

*Mantle = SWOG (adult); Arm B (adult) = ECOG 2496; Pediatric = COG AHOD 0031; n.s. = not significant*.

Table 4 compares the V5 for each organ for the mantle, Arm B and pediatric protocols. As expected, there is statistical difference between Mantle V5 (36 Gy) and Arm B (36 Gy) and the pediatric protocol (21 Gy), but surprisingly, no difference between Arm B at 36 Gy and pediatric at 21 Gy for V5 for each of the organs.

**Table 4:** **Heart, lung, and breast V5 analysis by protocol specified treatment prescription**.

	Arm B (36 Gy)	Pediatric (36 Gy)	Pediatric (21Gy)	SWOG (36 Gy)	Pediatric (36 vs. 21 Gy)	Arm B (36 Gy) vs. Ped. (21 Gy)	SWOG vs. Arm B (36 Gy)	SWOG vs. Ped. (21 Gy)
Heart (V5)	67.1	64.8	60.1	100	4.7	7	37.8	39.9
(95% Conf. Int.)	(53.0–81.2)	(50.6–78.9)	(46.0–74.2)	(85.9–114.1)	*p* = 0.9363 (ns)	*p* = 0.7719 (ns)	*p* < 0.0001	*p* < 0.0001
Lung (V5)	47.4	22.3	54.5	76.1	3.9	7.1	28.7	21.6
(95% Conf. Int)	(36.2–58.6)	(47.2–69.6)	(43.2–65.7)	(64.9–87.3)	*p* = 0.7614 (ns)	*p* = 0.2875 (ns)	*p* < 0.0001	*p* < 0.0001
Breast (V5)	25.1	33.4	30.5	49.6	2.9	−5.4	24.5	19.1
(95% Conf. Int.)	(12.6–37.7)	(20.8–45.9)	(17.9–43.1)	(37.1–62.2)	*p* = 0.9676 (ns)	*p* = 0.7561 (ns)	*p* < 0.0001	*p* = 0.0019

Table 5 examines the impact of dose reduction from 36 to 21 Gy within ECOG 2496 Arm B and the pediatric trials. It also compares RT doses for ECOG 2496 Arm B at 36 Gy to those for the pediatric trial at 21 Gy, which are the respective protocol specified doses. Dose reduction within ECOG 2496 Arm B from 36 to 21 Gy led to significantly lower doses to heart and lung but not breast. Similarly, dose reduction from 36 to 21 Gy within the pediatric trial led to lower doses to heart and lung but not breast. When ECOG 2496 Arm B 36Gy was compared with pediatric 21 Gy, the ECOG 2496 Arm B plan delivered significantly higher doses to the heart and lung tissue than the pediatric trial, but there was no significant difference in dosage delivered to breast.

**Table 5:** **Impact of dose reduction on normal tissue dose by treatment arm**.

	Arm B (36 Gy)	Arm B (21 Gy)	Pediatric (36 Gy)	Pediatric (21Gy)	Arm B (36 vs. 21 Gy)	Pediatric (36 vs. 21 Gy)	Arm B (36 Gy) vs. Ped. (21 Gy)
Heart (mean dose)	20.2	11.78	19.39	11.54	8.42	7.85	8.66
(95% Conf. Int.)	(15.47–24.92)	(7.05–16.51)	(14.66–24.11)	(6.82–16.27)	*p* = 0.0003	*p* = 0.0009	*p* = 0.0002
Lung (V20%)	35.09	21.42	42.75	26	13.67	16.75	9.09
(95% Conf. Int.)	(25.39–44.76)	(11.72–31.11)	(33.06–52.44)	(16.31–35.69)	*p* = 0.0004	*p* < 0.0001	*p* < 0.0001
Breast (mean dose)	7.01	4.09	8.52	5.21	2.92	5.6	1.8
(95% Conf. Int.)	(4.11–9.90)	(1.19–6.98)	(5.62–11.41)	(2.31–8.10)	*p* = 0.1257 (ns)	*p* = 0.045	*p* = 0.6356 (ns)

**Figure 4:**
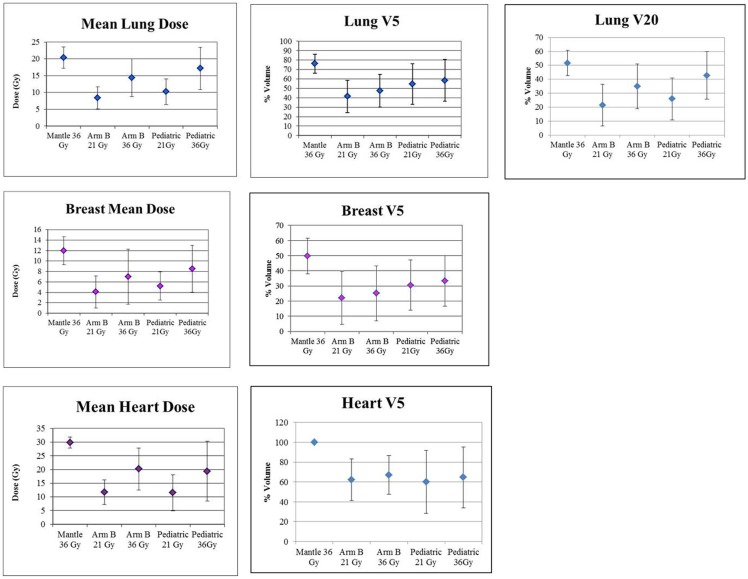
**Graphic presentation of lung, breast and heart mean dose and V5, and Lung V20. Error bars represent ± SD**.

Figure 4 graphically demonstrates mean and SD for V20 for lung and V5 and mean dose for each organ.

## Discussion

The majority of older adolescents and young adults, aged 16–21 years, were treated on pediatric protocols. A total of 15.2% were treated on adult protocols. According to the NCI, 2010 (15) adolescents and young adults have been more under-represented in clinical trials than children and middle-aged or older adults. Reasons include lack of access and referrals to specialized cancer centers and until recently, inadequate health insurance. The proportion of this age group treated off protocol using adult protocol regimens in community settings may be larger, but is not known. Community treatment is more likely to be administered by oncologists with greater experience with adult treatment regimens.

Treatment regimens that treat only involved fields, as defined on either adult (ECOG 2496 Arm B) or pediatric (COG AHOD0031) protocols, treat smaller volumes of normal tissue than traditional mantle fields (SWOG 9133). Furthermore, the protocol defined involved fields of the adult and pediatric protocols, although they seem quite different, would deliver the same dose to the normal structures, if the protocol specified doses were identical, either at 36 or 21 Gy. However, consistent with our original hypothesis, at the protocol prescribed dose of 36 Gy for adult trials and 21 Gy for pediatric trials, the adult trial delivered significantly higher doses to the heart and lung than the pediatric trial.

Of note, the hypothesis that breast tissue would receive significantly less RT dose on pediatric compared with adult trials was not fully supported (Table 5). The adult trial allowed axillary treatment only for nodes >5 cm while the pediatric protocol required axillary treatment for any nodal involvement. Only eight female patients were included in this study and only some received axillary treatment. Further investigation in larger samples is needed to evaluate if patients on pediatric protocols, with different field designs, receive a greater mean breast dose at 21 Gy than they would on adult regimens at 36 Gy.

In addition to small sample size, this study was also limited by the inclusion only of patients treated on these particular protocols. There is no way to know how many patients in this age group were treated off any protocol in this time period, or whether they were treated by adult or pediatric hematologists. And there is very limited outcome or late effects data on adolescents, because they are treated and followed on protocols combined with either adults or children.

Current National Cancer Cooperative Network (NCCN) Guidelines, 2014 (16) (which specifically exclude adolescents) recommend use of the lowest possible RT dose (range 20–36 Gy) and smallest volume for all HL patients based on bulk disease, stage, and CTX regimens. Furthermore, the guidelines recommend that axilla treatment is to be avoided in females if these regions are uninvolved.

However, questions remain on the optimal management of adolescents for long-term HL survival. Meyer et al. (17) recently found in a study of adults that patients have better long-term survival if treated without RT. However, their study randomized only patients with non-bulky stage IA and IIA disease to subtotal nodal radiation (35 Gy) or not. All these patients would have been excluded from RT on either the ECOG or pediatric trials. For local control, Wolden et al. (18) demonstrated significantly decreased 10-year event free survival in pediatric patients enrolled on study CCG 5942 who were “spared” RT, although there was no difference in overall survival at 10 years. IFRT requires treatment of the entire nodal region but the definition of involved field has been shrinking.

One limitation of this study is that it compared dosimetry of recently completed protocols, which had accrued patients between 1992 and 2010, and whose design dates back to the 1980s. During the past quarter century, our approach for both adults and children to the combination of radiation and CTX in HL has undergone dramatic changes in treatment target, dose, and technique with the aim of reducing the risk of damage to critical normal tissues.

The first strategy is a reduction of the radiation target volume from one targeting both known nodal involvement sites and elective volumes to one targeting only initially involved nodes. This technique of involved node radiation therapy was originally described by investigators from Vancouver and Germany (19, 20) and more recently has been codified in publications from the international lymphoma radiation oncology group (ILROG) (21). The ILROG has suggested both involved node RT when patients can have planning CT/PET scans done in treatment position prior to the start of CTX and a slightly broader approach termed “involved site RT” then this information is not available. Both approaches target only those nodes known (by virtue of enlargement or FDG uptake) on pre-CTX imaging, do not treat nodal “fields” or other elective volumes.

A second approach reduces the dose to the targeted volume, typically from the doses initially used when using radiation as a single modality of 36–44 Gy to doses in the range of 20–30 Gy. Such straightforward reductions in irradiated volume and high-dose volume would be expected to reduce the risk of both second malignancies and late cardiovascular damage. At least for adults, the best data for reductions to 20 Gy are for a carefully selected group of very favorable patients (stage I–IIA, no more than two sites of disease, no masses >5 cm) and extrapolation of the regimen of two cycles of ABVD and 20 Gy to patients with more advanced disease are not well supported (22).

A third technique has been to switch from the older AP/PA beam arrangements, which were typical of HL treatment through the 1990s to more conformal beam arrangements, typically using IMRT or VMAT. A number of groups have published data on the reductions in dose to critical normal tissue (e.g., breast and heart) while achieving high conformality around the target volume, which can be achieved with these techniques, both for patients with typical extent of disease and those with bulky mediastinal adenopathy (23, 24). This gain in conformality of the high-dose volume is, however, typically achieved by treating much larger volumes of surrounding tissue to lower doses. While clearly beneficial in reducing acute toxicity the implications of this for late effects particularly carcinogenesis in breast and lung (25) are uncertain and estimates of risk have been highly dependent on the model used (e.g., linear vs. non-linear) for predicting cancer risk in clinically relevant dose ranges (26, 27). The decision between AP/PA, conformal, and IMRT/VMAT treatments should be made on a case-by-case basis after careful review of plans generated with each of these techniques and may entail a discussion with the patient of tradeoffs between early vs. late treatment toxicities (28).

The next steps under investigation in children and adults include use of metabolic response driven treatment adaptation, potentially allowing further decrease in radiation treatment volume (4, 5) and use of protons, further decreasing treatment volume (29). There is concern about and neutron contamination from protons possibly increasing late carcinogenesis while reducing acute toxicity (30, 31). If possible, future studies should report on adolescents separately.

Late effects, such as secondary malignant neoplasms (SMNs) (31) and cardiovascular disease (32), may seriously impact long-term survival (33), using a pediatric model, Maraldo et al. determined that decreased field size and RT prescription dose were associated with a lower estimated risk of late effects and better survival. Similarly, Yeh and Diller (34) examined the trade-offs between short- and long-term mortality risks associated with RT in a hypothetical cohort of pediatric (15 years old) HL patients. Disease-specific lifetime mortality risk was lower with chemoradiotherapy (2%) compared with CTX alone (3.6%). However, the risk of excess late-effects mortality was lower with CTX alone (1.8%) compared with chemoradiotherapy (7.4%). Overall conditional life expectancy was 57.2 years with CTX alone compared with 56.4 years with chemoradiotherapy, suggesting that initial single modality treatment may provide better overall survival. For children and adolescents, many decades of follow-up are required, especially if event free survival is being regarded as questionable surrogate for long-term overall survival in HL.

To better understand the impact of late effects, Travis et al. (35) recently recommended research priorities in assessing interactions between RT and numerous confounding factors such as age, sex, race, substance use, diet, and genetic susceptibility with a focus on adolescents and young adults. For example, should treatment be altered for the young woman who has a strong family history of breast cancer with or without known BRCA mutation or the young man whose father died of a myocardial infarction while jogging at age 45?

In conclusion, adolescents treated on this adult protocol would have received higher RT dose to heart and lung compared to those on this pediatric protocol. The effect on breast dose was less clear and requires further study, with greater statistical power. Nevertheless, any radiation exposure requires careful records of the details of treatment, especially RT dose and field design, and very long-term follow-up for local control, late effects, and overall survival. This paper suggests that careful records of DVHs for normal organs should be kept for late effect analysis for every patient, on or off protocol. Until more research is available, adolescents and young adults should be treated on cooperative group protocols to the extent possible, and if not, an off protocol treatment regimen for each HL patient should be designed on a case-by-case basis with attention to field design, RT dose, and factors that may exacerbate late effects based on detailed medical history, to maximize long-term survival and quality of life.

## Conflict of Interest Statement

This study was supported by NIH/NCI Grant CA029511. None of the authors have any other financial conflicts to declare.
